# 20(S)-Ginsenoside Rg3 Promotes HeLa Cell Apoptosis by Regulating Autophagy

**DOI:** 10.3390/molecules24203655

**Published:** 2019-10-10

**Authors:** Shuai Bian, Yue Zhao, Fangyu Li, Shuyan Lu, Siming Wang, Xueyuan Bai, Meichen Liu, Daqing Zhao, Jiawen Wang, Dean Guo

**Affiliations:** 1 Jilin Ginseng Academy, Changchun University of Chinese Medicine, Changchun, Jilin 130117, China; 18843449574@163.com (S.B.); zy1234569515@163.com (Y.Z.); L18843926718@163.com (F.L.); lushuyan1996@163.com (S.L.); lwsm126030@126.com (S.W.); baixy1212@163.com (X.B.); liumc0367@163.com (M.L.); zhaodaqing1963@163.com (D.Z.); 2 Shanghai Research Center for Modernization of Traditional Chinese Medicine, National Engineering Laboratory for TCM Standardization Technology, Shanghai Institute of Materia Medica, Chinese Academy of Sciences, Shanghai 201203, China

**Keywords:** 20(*S*)-ginsenoside Rg3, autophagy, apoptosis, HeLa cells

## Abstract

20(*S*)-Ginsenoside Rg3 (GRg3) has various bioactivities including anti-cancer effects and inhibition of autophagy. However, no reports have investigated the appearance of autophagy or the connection between autophagy and apoptosis in HeLa cells treated with 20(*S*)-GRg3. Cell viability was measured by CCK-8 (cell counting kit-8) assays. Apoptosis and the cell cycle were analyzed by Hoechst 33342 staining and flow cytometry. Apoptotic pathways were examined by ROS (reactive oxygen species) determination and rhodamine 123 assays. Western blot analysis was used to determine changes in protein levels. Autophagy induction was monitored by acidic vesicular organelle staining and EGFP-LC3 transfection. 20(*S*)-GRg3 inhibited autophagy of cells in a starved state, making it impossible for cells to maintain a steady state through autophagy, and then induced apoptosis. 20(*S*)-GRg3 blocked the late stage of autophagy (fusion of lysosomes and degradation of autophagic lysosomes), including a decrease in acidic vesicular organelle fluorescence, increased LC3 I–II conversion, accumulation of EGFP-LC3 fluorescence, GFP-mRFP-LC3 red-green fluorescence ratio, degradation of the substrate p62, and loss of the balance between autophagy and apoptosis, which induced apoptosis. ROS increased, the mitochondrial membrane potential decreased, apoptotic inducer AIF was released from mitochondria, and nuclear transfer occurred, triggering a series of subsequent apoptotic events. Autophagy inducer rapamycin inhibited the apoptosis induced by 20(*S*)-GRg3, whereas autophagy inhibitor BA1 promoted apoptosis induced by 20(*S*)-GRg3. Therefore, 20(*S*)-GRg3 promoted HeLa cell apoptosis by regulating autophagy. In the autophagic state, 20(*S*)-GRg3 can be used as a novel autophagy inhibitor in synergy with tumor-blocking therapies such as chemotherapy, which supports its application in the medical field.

## 1. Introduction

Cervical cancer is the fourth most common cancer affecting women worldwide, especially in less developed regions. There are about 528,000 new cases each year, accounting for 7.5% of all cancer deaths of females [1,2]. Cervical cancer is associated with the human papilloma virus (HPV) [3]. It is a serious health threat to women, and there are many causes for high risks of HPV infection, such as a high number of sex partners, early sexual activity, and immunosuppression by human immunodeficiency virus [4]. HPV infections are usually short and asymptomatic. In the early stages of cervical cancer, women may not show any symptoms. The main treatments for cervical cancer are radiotherapy and chemotherapy [5]. Cancer cells undergo some fundamental changes from normal cells. Unlimited proliferation is one of the most fundamental biological behaviors of tumor cells, which is caused by alterations in multiple genes and their regulation [6]. Plant-derived compounds are characterized by low toxicity and have a wide range of anti-cancer activities, which are promising as new anti-cancer therapies [7].

*Panax ginseng* C.A. Meyer is a well-known traditional medicinal plant that has been widely used in Asian regions for thousands of years and has recently become increasingly popular in western countries [8]. In traditional Chinese and Korean medicine, ginseng is used for the treatment and prevention of cancer, diabetes, obesity, and cardiovascular diseases [3,8]. *Panax ginseng* C.A. Meyer is commonly consumed as a traditional health functional food [3,9]. Ginsenosides are among the most important active components in ginseng. 20(*S*)-GRg3 has numerous pharmacological effects on humans, including immunomodulatory effects [10] as well as anti-cancer effects against ovarian cancer [11], hepatocarcinoma [12], and human osteosarcoma [13].

Autophagy is a vital metabolic process in eukaryotic cells, which captures, degrades, and recycles intracellular components including aggregated proteins, soluble proteins, organelles, and macromolecular complexes in lysosomes. It prevents the accumulation of toxic proteins, preserves organelle functions, and promotes cell survival under starvation and stress conditions. Autophagy plays significant roles in the regulation of cell survival and death, especially apoptosis signaling pathways [10,14,15,16]. Some researchers view both stimulation and inhibition of autophagy as treatments for cancer [9].

The effects of various ginsenosides on autophagy have been investigated, and recent reports have shown that 20(*S*)-ginsenoside Rh2 inhibits autophagy and promotes apoptosis in human acute lymphoblastic leukemia cells [17]. Ginsenoside compound K induces autophagy to sensitize human colon cancer cells [18]. 20(*S*)-GRg3 induces autophagy to eliminate prion protein-mediated neurotoxicity and mitochondrial damage [19].

In the present study, we investigated the inhibitory effect of 20(*S*)-GRg3 on autophagy and whether it is functionally related to apoptosis in the absence of serum. Our data show that 20(*S*)-GRg3 inhibits autophagic flux by suppressing late stage autophagosome maturation or degradation. Because a lack of serum induces autophagy that has a prosurvival function, 20(*S*)-GRg3-induced suppression of autophagy contributes to apoptosis of HeLa cells. These findings provide a significant basis to elucidate the mechanisms by which the inhibitory effect of 20(*S*)-GRg3 on autophagy is functionally related to starvation-induced apoptosis, and may support the clinical application of 20(*S*)-GRg3.

## 2. Results

### 2.1. 20(S)-GRg3 Inhibits the Proliferation of Serum-Starved HeLa Cells

**Figure 1:**
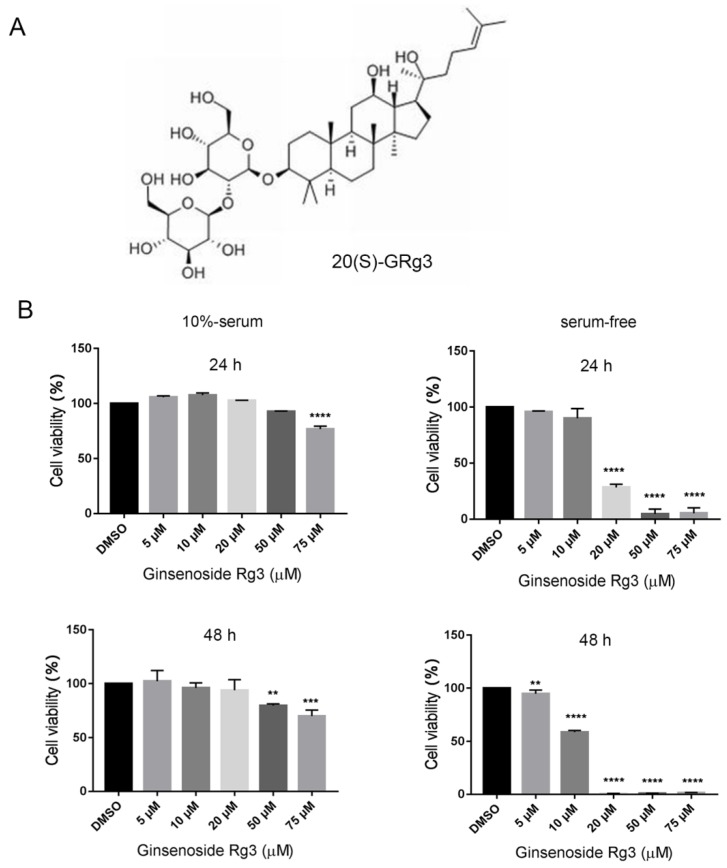
20(*S*)-GRg3 inhibits the proliferation of serum-starved HeLa cells. (**A**) Chemical structure of 20(*S*)-GRg3. (**B**) Effects of treatment with 20(*S*)-GRg3 at various concentrations and different times (24/48 h). ** *p* < 0.01, *** *p* < 0.001, **** *p* < 0.0001.

To determine whether 20(*S*)-GRg3 inhibits HeLa cell proliferation, we exposed the cells to 20(*S*)-GRg3 at different concentrations for various times, and then assessed cell viability. The data revealed that 20(*S*)-GRg3 notably suppressed HeLa cell proliferation in dose and time-dependent manners (Figure 1B). Under normal culture conditions, the IC_50_ values of 20(*S*)-GRg3 in HeLa cells was 50 µM with a treatment time of 24 h and 75 µM, with a treatment time of 48 h. In the absence of serum, the IC_50_ values of 20(*S*)-GRg3 in HeLa cells were 25 µM with a treatment time of 24 h and 5 µM, with a treatment time of 48 h. These results showed that, in the absence of serum, 20 µM 20(*S*)-GRg3 treatment for 24 h had strong anti-tumor activity.

### 2.2. 20(S)-GRg3 Increases Apoptosis and Regulates the Cell Cycle

**Figure 2:**
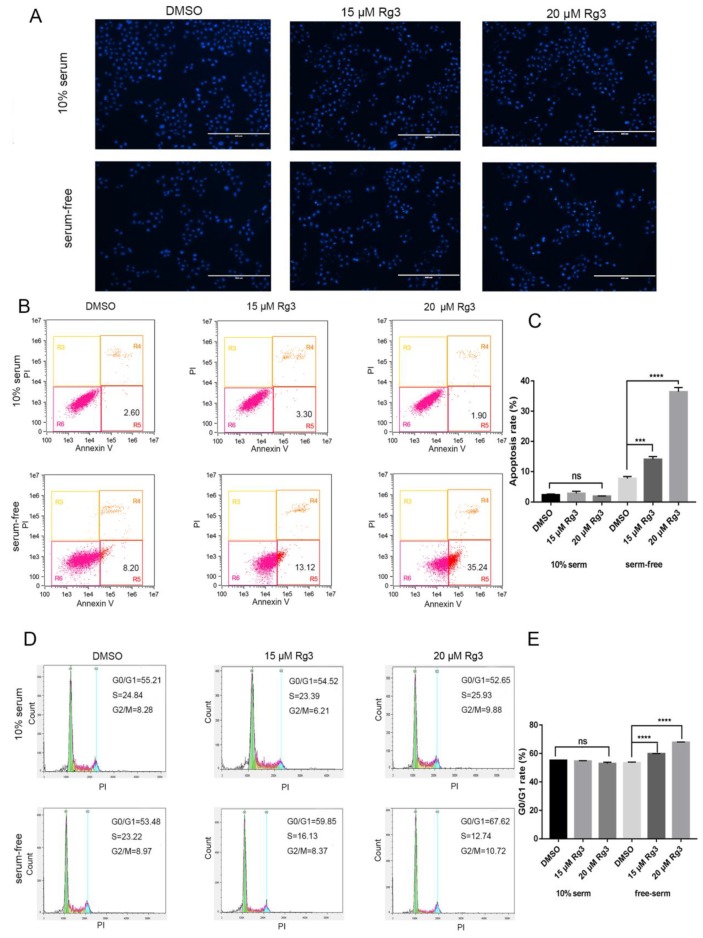
20(*S*)-GRg3 increases apoptosis and regulates the cell cycle. (**A**) Hoechst 33342 staining of Hela cells treated with 20(S)-GRg3 for 24 h. Apoptosis was characterized by chromatin condensation and nuclear fragmentation. (**B**) Flow cytometric analysis of the apoptosis ratio. The total apoptotic cell ratio was the sum of cells undergoing early apoptosis (lower right) and late apoptosis (upper right). Control cells received an equal volume of 0.1% DMSO. (**C**) Statistical analysis of apoptosis determined by the flow cytometric evaluation. (**D**) Flow cytometric analyses of the cell cycle distribution of Hela cells treated with different 20(*S*)-GRg3 concentrations under normal or serum-deprived conditions for 24 h. (**E**) Statistical analysis of the cell cycle distribution. *** *p* < 0.001, **** *p* < 0.0001, ns > 0.05.

To determine whether the decrease in cell viability was caused by apoptosis, we implemented Hoechst 33342 and annexin V/PI staining for cell cycle distribution analysis. The results showed that 20(*S*)-GRg3 did not affect apoptosis of tumor cells growing under normal culture conditions. However, in the absence of serum, high chromatin condensation and nuclear fragmentation were observed in HeLa cells treated with 20(*S*)-GRg3 dose-dependently (Figure 2A). Flow cytometry was used to distinguish between different stages of apoptosis as well as necrosis by the annexin V/PI assay. The total apoptotic rate was evaluated by the sum of early and late apoptotic cell proportions. In the absence of serum, 20(*S*)-GRg3 treatment resulted in apoptosis ratios of 13.12% (15 µM) and 35.24% (20 µM), indicating increased apoptosis of HeLa cells in a dose-dependent manner (Figure 2B). 20(S)-GRg3 did not affect the distribution of HeLa cells cultured in normal medium. However, 15 and 20 µM 20(*S*)-Rg3 increased the G0/G1 cell ratio (Figure 2D). These data suggested that 20(*S*)-Rg3 increased apoptosis and regulated the cell cycle in the absence of serum.

### 2.3. 20(S)-GRg3 Induces Apoptosis through the Mitochondrion-AIF Pathway

**Figure 3:**
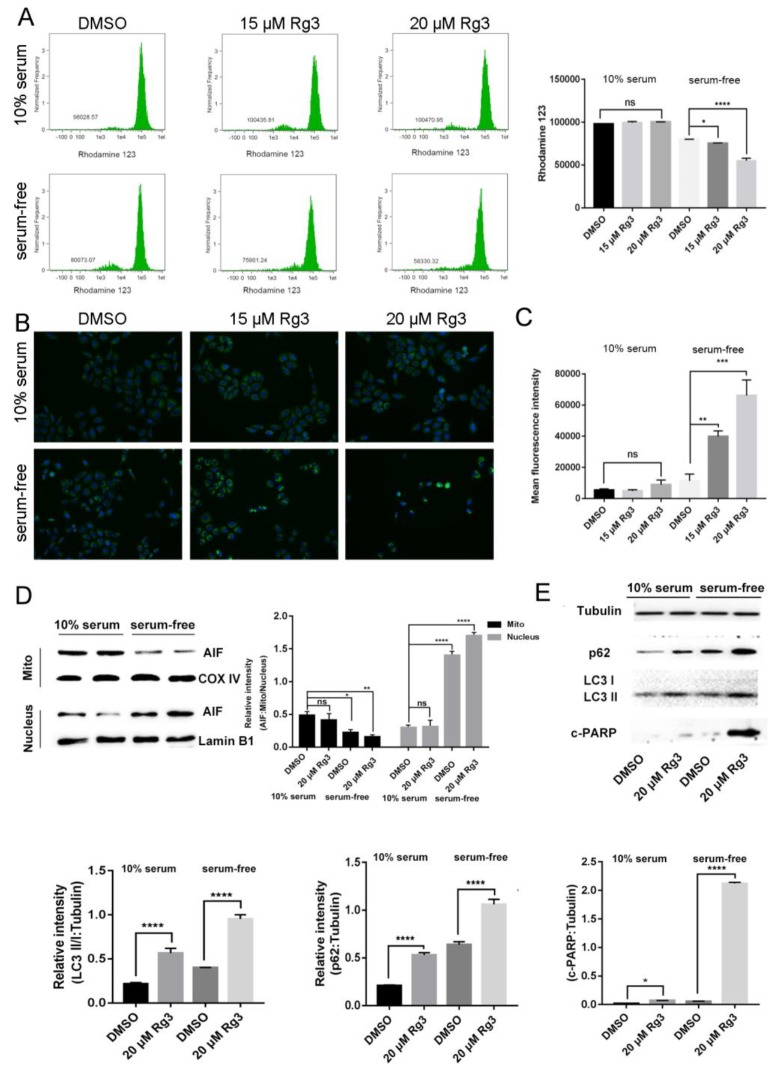
20(*S*)-GRg3 induces apoptosis through the mitochondrion-AIF pathway. (**A**) Flow cytometry in combination with rhodamine 123 staining was performed in Hela cells. Hela cells were treated with DMSO or 20(*S*)-GRg3 in the presence or absence of serum. After 24 h, cells were harvested and subjected to rhodamine 123 staining followed by flow cytometry. (**B**) Immunofluorescence analysis of AIF (green) in Hela cells. Hela cells were treated with DMSO or 20(*S*)-GRg3 in the presence or absence serum. Then, the cells were fixed for immunofluorescence with an anti-AIF antibody. DAPI was used for nuclear staining. (**C**) Production of intracellular ROS detected by the H2DCFDA fluorescent probe. (**D**) Subcellular distribution of AIF in Hela cells detected by western blotting. HeLa cells were treated with DMSO or 20(*S*)-GRg3 in the presence or absence of serum for 24 h and then collected for subcellular fractionation. (**E**) HeLa cells were treated with DMSO or 20(*S*)-GRg3 in the presence or absence of serum and harvested after 24 h. The proteinexpression of p62, LC3 and c-PARP were evaluated by a western blotting assay. * *p* < 0.05, ** *p* < 0.01, *** *p* < 0.001, **** *p* < 0.0001, ns > 0.05.

To investigate whether 20(*S*)-GRg3 enhances apoptosis without serum through the mitochondrion-AIF pathway, we compared ROS in control and 20(*S*)-GRg3 groups (Figure 3C). ROS destroys the mitochondrial membrane, changes the membrane permeability, and internal and external ions in the membrane reduce the concentration difference through free diffusion, thereby leading to a decrease in membrane potential. MMPs (mitochondrial membrane potential) were compared between the control group treated with rhodamine 123 and the 20(*S*)-GRg3 group. Flow cytometric analysis showed that 20(*S*)-GRg3 had little effect on the mitochondrial membrane potential under normal culture conditions, but significantly reduced MMP in serum-deprived cells (Figure 3A). These data suggest that 20(*S*)-GRg3 promotes mitochondrial depolarization in serum-deprived cervical cancer cells. Next, we detected the protein distribution of AIF. As shown in Figure 3B, the level of AIF in the nucleus of cells treated with 20(*S*)-GRg3 was significantly higher than that in the DMSO control group under serum deprivation. Moreover, AIF nuclear accumulation was accompanied by a decrease in mitochondrial distribution, indicating that 20(*S*)-GRg3 enhanced AIF release and nuclear translocation in serum-starved cells. In addition, immunofluorescence analysis of AIF showed that AIF accumulated more in the nucleus of 20(*S*)-GRg3-treated cells under serum deprivation than in cells under other conditions (Figure 3D). Western blotting showed that LC3-II and p62 protein levels were increased after 20(*S*)-GRg3 treatment. It indicated that 20(*S*)-GRg3 affects autophagy of HeLa cells. Western blotting of apoptosis-related proteins suggested that 20(*S*)-GRg3 induced HeLa cell apoptosis by changing the protein level of c-parp(Figure 3E). These data suggest that 20(*S*)-GRg3 promotes apoptosis under serum deprivation through the AIF pathway.

### 2.4. 20(S)-GRg3 Represses Starvation-Induced Autophagic Flux

**Figure 4:**
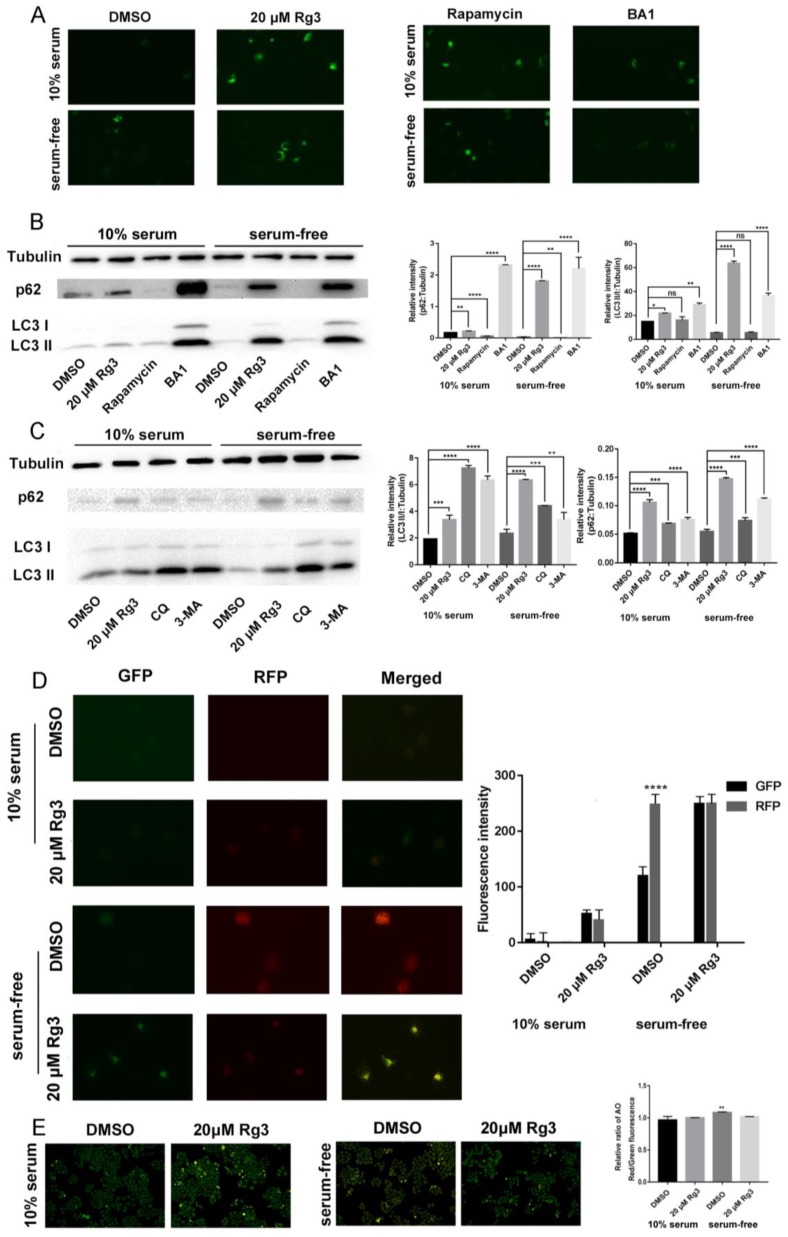
20(*S*)-GRg3 represses starvation-induced autophagic flux. (**A**) Hela cells were transfected with GFP-LC3 and then treated with DMSO, 20(*S*)-GRg3, 100 nM rapamycin, or 100 nM BA1 after 24 h in normal or serum-free media. After 24 h, the cells were observed under a fluorescence microscope. (**B**), (**C**) HeLa cells were treated with DMSO, 20(*S*)-GRg3, or autophagy regulators in the presence or absence of serum and harvested after 24 h. The proteinexpression of p62 and LC3 were evaluated by a western blotting assay. (**D**) HeLa cells were transiently transfected with GFP-mRFP-LC3 and cells were treated with DMSO or 20(*S*)-GRg3 in the presence or absence of serum and harvested for 24 h. At the end of treatment, cells were observed for the change of both green and red fluorescence using a confocal microscope. (**E**) Acidic vesicular organelles were examined by incubating Hela cells with acridine orange followed by fluorescence microscopy. * *p* < 0.05, ** *p* < 0.01, *** *p* < 0.001, **** *p* < 0.0001, ns > 0.05.

To determine whether 20(*S*)-GRg3 induced autophagy, the pEGFP-LC3 plasmid was transiently transfected into HeLa cells to confirm autophagy. 20(*S*)-GRg3 treatment markedly increased EGFP-LC3 puncta formation in HeLa cells (Figure 4A), confirming that 20(*S*)-GRg3 induced autophagy. Similar to rapamycin, 20(*S*)-GRg3 significantly increased the number of LC3 spots in HeLa cells cultured under normal and starvation conditions.

The increase of LC3-II induced by 20(*S*)-GRg3 may represent either increased generation of autophagosomes and/or a blockade of autophagosomal maturation and degradation [20,21,22,23]. The multifunctional cargo protein p62 binds to LC3 and is degraded with its cargo within autolysosomes [24]. Thus, a reduction of p62 can be regarded as a marker for an increase in autophagic flux [22,25]. LC3 and p62 protein levels were detected in 20(*S*)-GRg3-treated cells. Western blotting showed that LC3-II and p62 protein levels were increased after 20(*S*)-GRg3 treatment. Rapamycin induces autophagy by decreasing p62 protein levels, suggesting promotion of autophagy. However, the LC3-II protein level was not significantly increased, possibly because of the long treatment time of rapamycin, leading to LC3-II depletion. Autophagy inhibitor BA1, CQ and 3-MA prevented the maturation of autophagic vesicles by inhibiting autophagosome/lysosomal fusion and increasing LC3 and p62 levels (Figure 4B,C). 20(*S*)-GRg3 treatment of HeLa cells transiently transfected with tandem labeled GFP-mRFP-LC3, suggesting that upon 20(*S*)-GRg3 treatment the autophagosomes do not fuse with lysosomes or that lysosomal function is impaired (Figure 4D). In contrast, induction of autophagy by nutrient starvation led to the production of large amounts of red-only puncta, which blocks lysosomal function, indicating that 20(*S*)-GRg3 prevents autophagic flux, and does not increase it (Figure 4D).

Because the autophagic flux depends on low pH, we analyzed the lysosomal pH by an acridine orange (AO) assay. AO is a fluorescent nucleic acid dye that accumulates in acidic spaces such as lysosomes. Under the low pH conditions in lysosomes, the dye emits red light when excited by blue light.

Compared to the control group with serum, the DMSO group without serum showed an increased red signal, indicating that the pH value was decreased and autophagy was promoted, whereas the addition of 20(*S*)-GRg3 reduced the red signal, indicating inhibition of autophagy (Figure 4E). These data suggest that the increase in the LC3 II-level and EGFP-LC3 puncta formation of 20(*S*)-GRg3-treated cells was not due to the enhancement of autophagy, but the inhibition of autophagy in the late maturation or degradation stages, leading to autophagy flux blockade.

### 2.5. Apoptosis Induced by 20(S)-GRg3 is Associated with Autophagy

**Figure 5:**
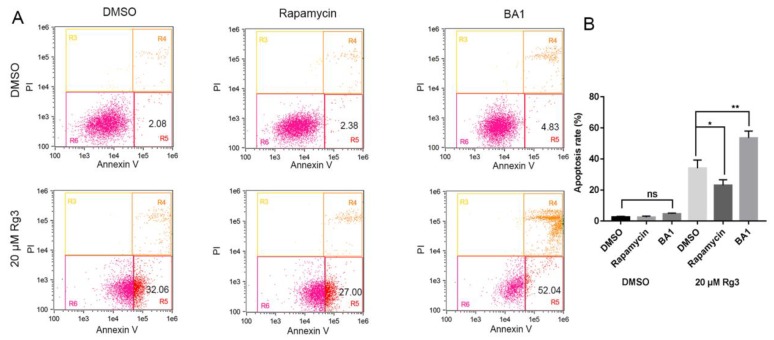
Apoptosis induced by 20(*S*)-GRg3 is associated with autophagy. (**A**) Flow cytometric analyses of apoptotic Hela cells treated with DMSO or G-Rg3 under serum-free conditions in the presence or absence of 100 nM BA1 or 100 nM rapamycin. (**B**) Statistical analysis of apoptosis determined by the flow cytometric evaluation. * *p* < 0.05, ** *p* < 0.01, ns > 0.05.

To determine whether autophagy regulation was the cause of apoptosis induced by 20(*S*)-GRg3, we used two autophagy regulators, the inhibitor bafilomycin A1 (BA1) and inducer rapamycin, to regulate autophagy. Figure 5 shows that, in the absence of serum, the apoptosis rate of HeLa cells induced by 20(*S*)-GRg3 was increased by BA1 treatment and decreased by rapamycin treatment. These data suggest that autophagy plays an important role in 20(*S*)-GRg3-induced apoptosis under serum-deprived conditions.

## 3. Discussion

As a global health problem, there has been a rise in the morbidity and mortality of cancer in recent years. Botanical medicines have been used to treat various diseases in Asia for thousands of years. Ginseng is one of the most well-known and widely used oriental medicinal plants. After being dried, steamed, and heated, ginseng is distributed in 35 countries in various forms [26]. Several recent studies have reported the beneficial effects of ginseng on diseases such as cancer, immune disorders, diabetes, and liver, nervous system, cardiovascular, and infectious diseases [27,28,29,30,31,32]. Ginsenoside affects various signaling pathways, and its effect on autophagy is unclear. In this study, we provide evidence that 20(*S*)-GRg3 has an inhibitory effect on autophagy and promotes apoptosis by inhibiting autophagy in HeLa cervical cancer cells. Therefore, 20(*S*)-GRg3 may have the potential to be developed as a novel cancer drug to inhibit the autophagic ability of tumor cells. 20(*S*)-GRg3 is a new autophagy inhibitor with potential clinical and research value. It can be used in combination with nutritional blockade (interventional therapy), or chemotherapeutic drugs that cause autophagy in treated tumors.

The concept of apoptosis was first introduced in 1972 and known as programmed cell death [33]. The mechanism of apoptosis is very complex and involves an energy cascade of molecular events [34,35]. Targeted apoptosis is the most successful non-surgical treatment among tumor treatment methods. We found that in the absence of serum, the IC50 values of 20(*S*)-GRg3 in HeLa cells were 20 µM with a treatment time of 24 h. The apoptosis induced by 20(*S*)-GRg3 was associated with autophagy, which was accompanied by prominent increases in condensed nuclei, an increase in the apoptosis rate, and change in the cell cycle. 20(*S*)-GRg3 caused mitochondrion-mediated apoptosis, ROS production, mitochondrial membrane degradation, apoptosis-inducing factor AIF was released from mitochondria, and nuclear translocation, which induce a series of subsequent apoptotic events.

In terms of the effect of 20(*S*)-GRg3 on apoptosis of HeLa cells under the conditions of autophagy, because autophagy is a necessary metabolic process to maintain cell survival in the state of starvation, 20(*S*)-GRg3 inhibited this metabolic process of cells, making it impossible for cells to maintain homeostasis through autophagy, and then induced apoptosis.

Autophagy is a catabolic process that is conserved across all eukaryotes. From regulating basic metabolic functions in cells to various pathological conditions, autophagy has become the central regulation point to control human homeostasis [36]. Under serum-free conditions, 20(*S*)-GRg3 induced apoptosis, and significantly increased the LC3 I to LC3 II conversion and the combination of GFP-LC3-II and autophagosome membranes. 20(*S*)-GRg3 inhibited late autophagy by inhibiting maturation, fusion, or degradation. Although the molecular mechanism underling the effect of 20(*S*)-GRg3 on autophagic flux remains to be studied further, the effect of 20(*S*)-GRg3 on autophagy may be similar to that of BA1 by interfering with lysosome functions. 20(*S*)-GRg3 inhibits autophagic flux in the late stage of autophagy (fusion of autophagosomes and lysosomes or degradation of autophagosomes). After the balance between autophagy and apoptosis was lost, apoptosis was initiated.

## 4. Materials and Methods

### 4.1. Antibodies and Reagents

The following antibodies were used in this study: mouse anti-tubulin mAb (BioLegend, San Diego, CA, USA), rabbit anti-Cleaved PARP mAb, rabbit anti-LC3A/B mAb, mouse anti-p62 mAb, (Cell Signaling Technology, Danvers, MA, USA), and HRP-conjugated goat anti-mouse and anti-rabbit IgGs (Jackson Immunoresearch, West Grove, PA, USA).

20(*S*)-GRg3 (Figure 1A) was purchased from Chengdu Must Biotech (Chengdu, China). An Annexin V-FITC/PI apoptosis detection kit was purchased from BD Biosciences (Franklin Lakes, NJ, USA). Rhodamine 123, Hoechst 33342, a ROS detection assay kit, and Braford assay kit were purchased from Beyotime. Cell counting kit-8 (CCK-8) was purchased from BOSTER Biotech (Wuhan, China). Rapamycin, 3-Methyladenine (3-MA), Chloroquine diphosphate (CQ) and bafilomycin A1 (BA1) were purchased from InvivoGen.

### 4.2. Cell Culture and Transfection

HeLa cells (ATCC, CCL-2) were cultured in DMEM with 10% fetal bovine serum at 37 °C in a 5% CO_2_ environment. Lipofectamine 2000 (Invitrogen, Carlsbad, CA, USA) was used for transient plasmid transfections.

### 4.3. Hoechst 33342 Staining Assay

After overnight culture in 12-well plates, cells were treated with 20(*S*)-GRg3 for 24 h. Cells were rinsed in phosphate buffered saline (PBS) and stained with Hoechst 33342 in the dark for 20 min. Then, the cells were observed at 405 nm under a fluorescence microscope and imaged.

### 4.4. CCK-8 Assay

Cells (3 × 10^3^) were cultured in 96-well plates overnight. Then, the culture supernatant was replaced with a medium containing dimethylsulfoxide (DMSO) or 20(*S*)-GRg3, and the cells were incubated for various times. For analysis, CCK-8 substrate was added to the 96-well plates, followed by incubation at 37 °C for 1 h. Absorbance was measured at 450 nm using a microplate reader (Tecan Infinite 200PRO, Tecan, Mannedorf, Switzerland).

### 4.5. Flow Cytometric Analysis of Apoptosis

After overnight culture in 6-well plates, cells were treated with DMSO or 20(*S*)-GRg3 for 24 h. The cells were digested, centrifuged, washed, and then double stained with annexin V-FITC and PI, in accordance with the manufacturer’s instructions. The samples were analyzed by a FlowSight^®^ Imaging Flow Cytometer (Merck Millipore, Burlington, MA, USA) and IDEAS Application V6.1 analysis software.

### 4.6. ROS Determination

After overnight culture in 6-well plates, cells were treated with DMSO or other compounds for 24 h. The cells were digested, centrifuged, washed, and then incubated with H_2_DCFDA (10 mM) for 30 min. The fluorescence of ROS was assessed by the FlowSight^®^ Imaging Flow Cytometer. Data were analyzed by IDEAS Application V6.1 (Andritz Inc., Decatur, GA, USA).

### 4.7. Rhodamine 123 Assay for Measurement of Mitochondrial Membrane Potentials

After overnight culture in 6-well plates, cells were treated with DMSO or other compounds for 24 h. The cells were digested, centrifuged, washed, and then stained with 1 µM rhodamine 123 for 30 min. Cells were rinsed with ice-cold PBS and analyzed by flow cytometry.

### 4.8. Acidic Vesicular Organelle Staining

After rinsing in PBS and fixing with 4% paraformaldehyde for 10 min, the cells were stained for acidic vesicular organelles in the dark for 30 min. Then, the cells were observed by fluorescence microscopy at 488 nm and imaged.

### 4.9. Western Blotting

Cells were harvested by centrifugation, resuspended with lysis buffer, and boiled for 15 min. Proteins were separated by SDS-PAGE and then transferred to nitrocellulose membranes (Whatman, Maidstone, UK). The membranes were blocked in 5% dry nonfat milk and then incubated with primary and secondary antibodies, successively. Immunoreactions were visualized by chemiluminescence.

### 4.10. Statistical Analysis

All data represent at least three independent experiments and are denoted as the mean ± SEM. The Student’s t-test was used for statistical comparisons. *p* < 0.05 was considered as statistically significant.

## 5. Conclusions

The incidence of cancer is increasing yearly. At present, the common treatment of most cancers is surgical resection combined with various chemotherapies, all of which have adverse effects and lead to drug resistance. Therefore, there is an urgent need for new and more effective treatment options for cancer. Because autophagy is usually a prosurvival response to therapeutic drugs, a combined therapy that inhibits autophagy during chemotherapy may be a good therapeutic strategy. Currently, chloroquine and its derivative hydroxychloroquine are the only drugs currently approved by the FDA as autophagy inhibitors [37]. Other clinical agents that inhibit autophagy are currently under development, but have not been approved for clinical use.

Inhibition of autophagy is a newly appreciated cellular regulatory mechanism, and the close relationship between autophagy and tumor development makes it of great significance to determine new therapeutic targets in tumors and the search for new anti-tumor drugs. Whether traditional Chinese medicine can achieve tumor inhibition by regulating autophagy of tumor cells is a new direction of anti-tumor research. However, the relationship between 20(*S*)-GRg3 and autophagy in tumor cells has not been clarified. In this study, we analyzed the regulation of autophagy by 20(*S*)-GRg3 and related activation/inhibition pathways, revealing the mechanism of ginsenoside 20(*S*)-GRg3-inhibited autophagy in cervical cancer cells and verifying the correlation between inhibition of autophagy and induction of apoptosis.

For a variety of cancers, including cervical cancer, interventional therapy is a novel therapy with fewer side effects and fewer complications [38]. Chemotherapy drugs can be locally targeted infusion. 20(*S*)-GRg3 plays an important role in the prevention and treatment of cancer [39]. It can be combined with other chemotherapy drugs through interventional therapy, which can avoid oral and intestinal degradation. Compared with oral administration, local application can reduce the dosage [40]. Vascular interventional therapy can be used, that is, the use of vascular blockers to reduce the tumor blood supplies [41]. Meanwhile, because 20(*S*)-GRg3 inhibits autophagy, it can be used in concert with anti-cancer drugs that cause autophagy of tumor cells [37].

20(*S*)-GRg3 can be used as a new autophagy inhibitor in combination with tumor-blocking therapy or other chemotherapies, which supports its application in the medical field.
